# Self-Ligating Brackets, Friction Reduction, Treatment Acceleration, and Patient-Centered Outcomes: A Review

**DOI:** 10.7759/cureus.111267

**Published:** 2026-06-21

**Authors:** Girish P Vasudeva Setty, Mohammad U Ahmed, Subash C Nayak, Sreeshma Sunder, Devanshi Churiwal, T Chandrasekhara Sastry, Kafeel Ahmed, Neena Bhatti

**Affiliations:** 1 Dentistry, Smile Architect Dental Care, Bengaluru, IND; 2 Orthodontics and Dentofacial Orthopedics, Dr. Ahmed Almousa Medical Centre, Al-Qassim, SAU; 3 Orthodontics, Hi-Tech Dental College & Hospital, Bhubaneswar, IND; 4 Orthodontics and Dentofacial Orthopedics, S Dental Care, Hyderabad, IND; 5 Dentistry, Rameshwar Prasad Golwara Memorial Hospital and Research Centre, Patna, IND; 6 Orthodontics and Dentofacial Orthopedics, Mamata Institute of Dental Sciences, Hyderabad, IND; 7 Periodontology, MNR Dental College and Hospital, Sangareddy, IND; 8 Pharmacology, Christian Medical College & Hospital, Ludhiana, IND

**Keywords:** accelerated orthodontics, friction reduction, patient-centered outcomes research, self-ligating brackets, sliding mechanism

## Abstract

Self-ligating brackets (SLBs) have emerged as an important innovation in contemporary orthodontics, offering the potential to minimize frictional resistance, improve biomechanical efficiency, and enhance patient-centered treatment outcomes. Unlike conventional brackets, which rely on elastomeric or metallic ligatures to engage the archwire, SLBs incorporate an inbuilt locking mechanism that permits freer wire movement and reduced binding during sliding mechanics. This narrative review examines the biomechanical principles, clinical efficacy, and future potential of SLB systems. Evidence supports several advantages, including reduced friction, improved alignment efficiency, shortened chairside time, better oral hygiene maintenance, and improved patient comfort. Reduced frictional resistance may also allow the use of lighter, more physiologic orthodontic forces, thereby promoting favorable periodontal responses. Despite strong in vitro evidence of improved mechanical performance, clinical trials and systematic reviews have reported mixed results regarding reductions in total treatment time and long-term superiority over conventional systems. The growing integration of digital orthodontics, AI, and personalized appliance technologies holds promise for further enhancing the clinical utility of SLBs. Large-scale randomized controlled trials with standardized methodology remain necessary to establish definitive conclusions on long-term efficacy.

## Introduction and background

**Figure 1:**
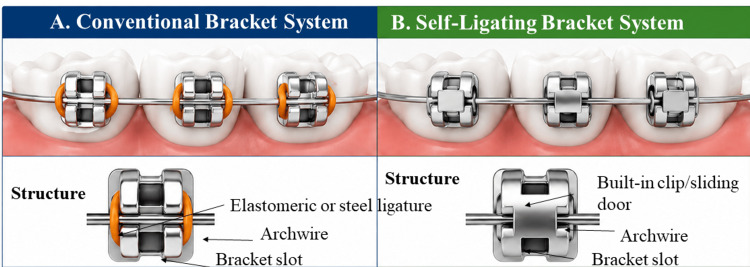
Comparison of conventional and SLB systems Schematic illustration comparing conventional orthodontic brackets, which require elastomeric or metallic ligatures for archwire engagement, with SLBs that incorporate an integrated clip or sliding mechanism to secure the archwire without external ligation. SLB, self-ligating bracket This figure was created by the authors using Microsoft PowerPoint (Microsoft Corporation, Redmond, WA, USA).

Orthodontic bracket systems have undergone considerable technological development over recent decades, driven by the need for improved biomechanical efficiency, shorter treatment durations, and enhanced patient comfort [1]. Traditional fixed appliances use elastomeric modules or stainless-steel ligatures to secure the archwire within the bracket slot; however, these ligatures generate frictional resistance that impedes sliding mechanics and may reduce tooth-movement efficiency. Sliding mechanics are defined as the movement of teeth along an archwire during alignment and space closure [2]. Self-ligating brackets (SLBs) address this limitation through an inbuilt locking mechanism that eliminates external ligatures and allows a more passive, low-friction interaction between the bracket and archwire (Figure 1) [3].

Self-ligation dates to the Russell attachment in the early 20th century and has since evolved into distinct active, passive, and interactive systems, including the SPEED, Damon, SmartClip, and In-Ovation appliances, each designed to optimize mechanical efficiency and clinical handling [4]. Active SLBs use a spring clip that presses against the archwire to enhance torque expression and rotational control, while passive brackets employ a slide that converts the slot into a tube-like channel, minimizing frictional resistance during alignment and leveling [3,5]. Torque expression is the ability of a bracket-wire system to control the buccolingual inclination and root position of a tooth. Interactive systems blend both mechanisms, adapting their force expression to wire size and treatment stage [4].

Frictional resistance at the bracket-archwire interface is a key factor influencing orthodontic tooth movement and treatment efficiency. Reduced frictional resistance has been proposed as one of the principal biomechanical advantages of SLBs, potentially facilitating more efficient sliding mechanics and improved force transmission during treatment [6,7]. In addition to mechanical considerations, contemporary orthodontic practice increasingly emphasizes periodontal health, treatment efficiency, and patient-centered outcomes, including comfort, aesthetics, and quality of life [8].

The absence of elastomeric ligatures in SLBs also reduces plaque-retentive surfaces, contributing to improved oral hygiene maintenance [9]. Patient-centered outcomes, including pain, chairside time, aesthetics, and quality of life, are increasingly recognized as key indicators of orthodontic success [10]. Their favorable biomechanical profile and patient-centered benefits have supported their continued adoption in contemporary orthodontic practice [3,5]. The key characteristics are compared in Table 1 [2-5].

**Table 1:** Comparison of key characteristics of conventional and self-ligating orthodontic bracket systems This table summarizes the principal differences between conventional and SLB systems with respect to archwire engagement, frictional resistance, force transmission, oral hygiene, patient comfort, treatment efficiency, and clinical handling. SLB, self-ligating bracket Adapted and synthesized by the authors from references [2-5]

Parameter	Conventional brackets	SLBs
Archwire engagement	Elastomeric ligatures or stainless-steel ties	Inbuilt clip or slide; no external ligatures
Frictional resistance	Higher due to ligature binding	Reduced, especially in passive systems
Bracket subtypes	Metal, ceramic, aesthetic ligated	Active, passive, and interactive
Force transmission	Greater force loss due to friction	More efficient force delivery
Chairside time	Longer ligation and adjustment time	Reduced; easier wire insertion and removal
Oral hygiene	Plaque accumulation around ligatures	Fewer plaque-retentive sites
Patient comfort	Higher friction contributes to discomfort	Often associated with reduced pain
Aesthetic options	Ceramic and aesthetic ligated systems	Aesthetic ceramic SLB systems available
Anchorage demand	May require greater anchorage reinforcement	Potential reduction in anchorage demand
Cost	Generally lower	Higher due to advanced bracket design
Treatment duration	Conventional duration	Some studies report faster alignment and leveling

This narrative review was conducted through searches of PubMed, Scopus, Google Scholar, and Web of Science. Literature retrieval was performed between January and March 2026. Search strategies combined controlled vocabulary and free-text terms using Boolean operators. Representative search strings included: ("self-ligating brackets" OR "self-ligating orthodontic appliances") AND ("orthodontics" OR "orthodontic treatment"), ("self-ligating brackets") AND ("friction" OR "sliding mechanics"), ("self-ligating brackets") AND ("treatment efficiency" OR "treatment duration"), and ("self-ligating brackets") AND ("periodontal health" OR "oral hygiene"). Preference was given to peer-reviewed articles published between 2021 and 2026, while seminal earlier studies were included when necessary to provide historical and biomechanical context. Articles were selected based on their relevance to bracket biomechanics, frictional resistance, treatment efficiency, periodontal outcomes, patient-centered measures, and emerging technologies in orthodontics.

## Review

Biomechanics and friction reduction

**Figure 2:**
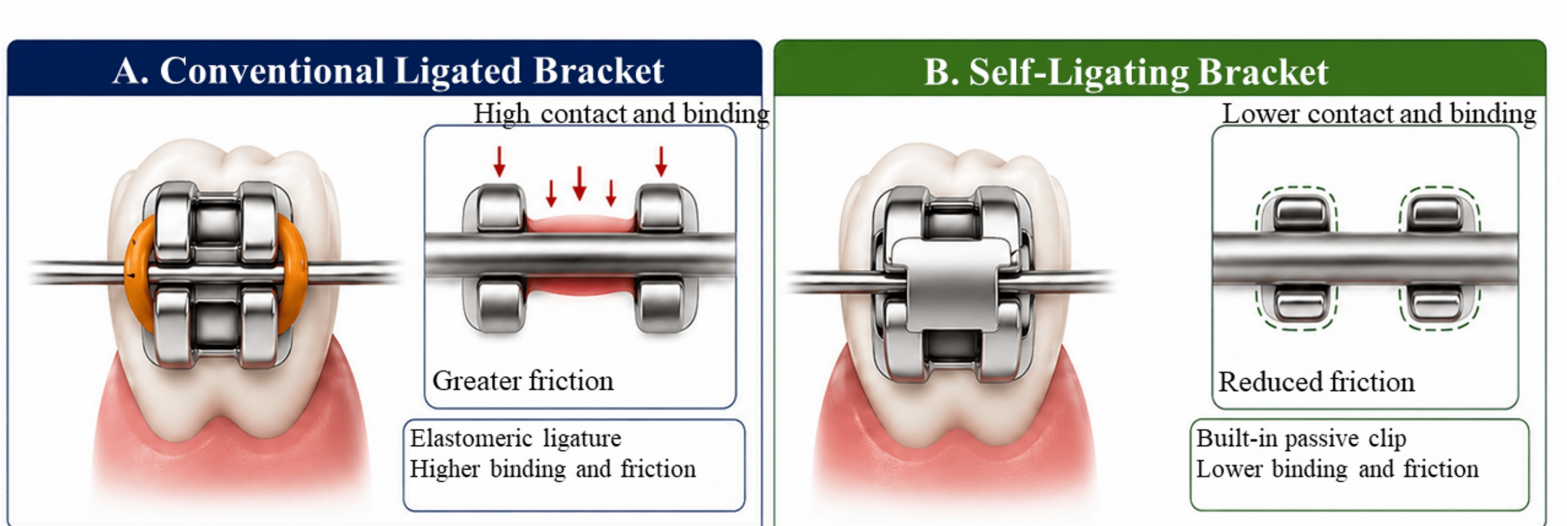
Biomechanical mechanism of friction reduction in SLBs Schematic representation of the biomechanical principles underlying friction reduction in SLBs. The figure illustrates how the elimination of external ligatures reduces binding and resistance to sliding, thereby facilitating freer archwire movement and more efficient force transmission during orthodontic tooth movement. SLB, self-ligating bracket This figure was created by the authors using Microsoft PowerPoint (Microsoft Corporation, Redmond, WA, USA).

Friction reduction is central to the proposed clinical superiority of SLBs [2]. In conventional systems, ligatures compress the archwire against the bracket slot, generating binding and notching forces that resist tooth movement [6]. SLBs replace this with a clip or slide mechanism that imposes minimal ligation force on the wire, allowing freer movement within the slot and substantially lower frictional resistance during alignment and space closure (Figure 2) [11,12].

Orthodontic friction comprises static, kinetic, and binding components, all of which are influenced by bracket design, ligation mode, wire size and material, bracket-wire angulation, surface roughness, and the oral environment [2,6]. In vitro experiments consistently demonstrate that passive SLBs produce markedly lower frictional forces than conventional brackets, especially with small nickel-titanium wires at low angulations; active SLBs may generate slightly higher friction owing to clip-induced wire loading [7]. These differences are clinically significant because reduced friction enables the delivery of lighter, more physiologic forces, promoting efficient tooth movement with less stress on periodontal tissues [5]. A detailed description is shown in Table 2 [2,6-8,11].

**Table 2:** Factors influencing frictional resistance and orthodontic treatment efficiency This table outlines the major mechanical and material-related factors that influence frictional resistance at the bracket-archwire interface and their potential effects on orthodontic tooth movement, anchorage control, and treatment efficiency. SLB, self-ligating bracket Adapted and synthesized by the authors from references [2,6-8,11]

Factor	Influence on friction	Effect on efficiency
Ligation mode	Passive clips reduce; elastomeric ligatures increase resistance	Fewer adjustments; smoother tooth movement
Bracket material	Ceramic brackets produce higher friction than stainless steel	May affect alignment rate and force delivery
Archwire material	Stainless steel produces lower friction than beta-titanium	Enhances sliding efficiency during space closure
Archwire dimension	Larger rectangular wires increase binding and notching	Greater torque control but higher resistance
Wire surface roughness	Rougher surfaces elevate frictional resistance	May delay tooth movement
Bracket slot accuracy	Inaccurate slot dimensions increase wire binding	Influences torque expression and control
Bracket-wire angulation	Higher angulation increases binding resistance	Reduces sliding efficiency
Oral environment	Saliva alters frictional behavior depending on materials	Clinical friction differs from in vitro measurements
SLB subtype	Passive systems generate lower friction than active systems	Facilitates smoother leveling and alignment
Anchorage control	Higher friction increases reactive forces on posterior teeth	May compromise anchorage stability

Superelastic nickel-titanium wires, used during initial alignment, benefit most from the low-friction environment of passive SLBs, while stiffer stainless-steel wires used in finishing and space closure also show improved sliding efficiency [12]. Anchorage control is directly influenced by friction, since higher frictional resistance amplifies reactive forces on posterior anchor teeth [2,11]. The reduced friction of SLBs may therefore mitigate anchorage loss, although clinical evidence of a meaningful advantage remains inconsistent [13]. The biological foundation of tooth movement, namely cyclic compression and tension of the periodontal ligament driving coordinated osteoclastic and osteoblastic activity, is best supported by controlled, physiologic force levels [14,15]. Force overload, compounded by excessive friction, disrupts vascular perfusion, promotes hyalinization, and increases the risk of root resorption and patient pain [16]. Modern orthodontic biomechanics, therefore, prioritizes friction reduction as a means of maintaining mechanical effectiveness without compromising periodontal health or patient comfort [8,11].

Clinical outcomes and patient-centered benefits

The principal clinical rationale for SLBs is improved treatment efficiency through reduced frictional resistance [3,5]. Smoother sliding mechanics are expected to facilitate faster tooth alignment and space closure, particularly in the early leveling phase [11]. Although some studies report faster alignment and fewer appointments, systematic reviews and meta-analyses have generally found insufficient evidence to support a clinically significant reduction in overall treatment duration compared with conventional bracket systems [3-5]. Pain and discomfort in orthodontic treatment stem primarily from inflammatory and vascular changes in the periodontal ligament following force application [16]. The lighter forces enabled by SLBs may reduce pain intensity during the early treatment phases, and the elimination of elastomeric ligatures decreases the risk of soft-tissue irritation and ligature-related plaque accumulation [9,10].

Periodontal health is a critical concern in fixed orthodontic treatment, as plaque retention around brackets and ligatures can cause gingival inflammation, enamel demineralization, and periodontal breakdown [17]. Conventional elastomeric ligatures have been associated with increased bacterial colonization and greater plaque retention than SLBs, which present smoother bracket surfaces and fewer retentive sites [18]. Recent systematic reviews confirm that SLBs show significantly lower plaque indices and reduced gingival inflammation compared with conventional systems, with periodontal outcomes consistently favoring SLBs across multiple clinical evaluations [19-21]. This aligns with evidence from recent systematic reviews confirming that self-ligating systems significantly lower the risk and prevalence of enamel demineralization and white spot lesions due to superior plaque management [9].

Beyond functional outcomes, esthetic and psychological factors play an important role in patient satisfaction. Ceramic SLB systems and low-profile bracket designs enhance treatment acceptance and reduce speech-related discomfort, contributing to improved quality of life during therapy [10]. Overall, current evidence suggests that SLBs provide practical clinical advantages, including improved chairside efficiency, oral hygiene maintenance, and patient comfort, while maintaining treatment outcomes comparable to conventional appliances (Table 3).

**Table 3:** Summary of recent clinical evidence evaluating SLBs This table summarizes key clinical studies, systematic reviews, meta-analyses, and RCTs assessing the effectiveness of SLBs compared with conventional bracket systems, with emphasis on treatment efficiency, periodontal outcomes, patient-centered benefits, and overall clinical performance. RCT, randomized controlled trial; SLB, self-ligating bracket

Author(s) and year	Study design	Sample	Comparison	Key findings	Clinical significance
Arora et al. (2025) [3]	Narrative review	Multiple RCTs and clinical studies	SLBs vs conventional: efficiency, comfort, hygiene	SLBs offer lower friction and better oral hygiene; no significant overall treatment-time advantage; patient comfort favored	Current best evidence supports patient-centered benefits over time savings
Baxi et al. (2023) [4]	Comprehensive review	Multiple studies	SLBs vs conventional brackets	Reported improved clinical efficiency and reduced chairside time; evidence regarding overall treatment duration remains inconsistent	Suggests practical efficiency benefits without definitive evidence of shorter overall treatment duration
Kambale et al. (2025) [5]	Systematic review	Multiple comparative studies	SLBs vs conventional: overall treatment efficacy	Comparable overall efficacy; chairside time and patient comfort advantages confirmed; no definitive treatment-time superiority	SLBs equally effective with added practical and comfort benefits
Idris et al. (2025) [9]	Systematic review	Multiple RCTs	SLBs vs conventional: white spot lesion incidence	White spot lesion prevalence lower in SLB patients than in conventional bracket patients	SLBs reduce enamel demineralization risk through better plaque management
Huang et al. (2025) [10]	Comparative clinical study	Conventional, SLB, and aligner groups	SLBs vs conventional vs aligners: pain, anxiety, oral health-related quality of life	SLBs associated with lower pain and anxiety scores than conventional brackets; improved oral health-related quality of life	SLBs improve patient-reported experience during treatment
Mester et al. (2022) [19]	Systematic review and meta-analysis	Six clinical studies	SLBs vs conventional periodontal outcomes	SLBs associated with lower plaque indices and reduced bleeding on probing compared with conventional brackets	SLBs offer a periodontal health advantage during treatment
Mukhopadhyay et al. (2022) [20]	Umbrella review (16 systematic reviews)	Multiple systematic reviews	SLBs vs conventional: overall outcomes	SLBs reduce mandibular incisor proclination, bacterial accumulation, chairside time, and oral malodor; no difference in overall treatment time	Confirms patient-centered and hygiene benefits; no treatment-time superiority
Hoyte et al. (2026) [21]	RCT	~50 patients (extraction)	SmartClip SLBs vs conventional: bimaxillary protrusion	No reduction in overall treatment time; fewer total visits in the SLB group; comparable quality of care	SLBs reduce appointment frequency without shortening total treatment duration
Coronel-Zubiate et al. (2024) [22]	Systematic review and meta-analysis	RCTs up to October 2022	SLBs vs conventional: periodontal health	No statistically significant difference in probing depth or gingival index; comparable periodontal health with good oral hygiene	Periodontal outcomes comparable when hygiene is maintained

Advantages, limitations, and future perspectives

SLBs offer several practical advantages, including simplified archwire engagement, reduced chairside time, improved oral hygiene maintenance, and enhanced patient comfort. The elimination of elastomeric ligatures reduces plaque-retentive surfaces and facilitates easier appliance management, while aesthetic SLB designs may further improve treatment acceptance and patient satisfaction [12,13,15].

Significant limitations remain, however, in the evidence base for overall clinical superiority. Despite favorable biomechanical properties and reduced frictional resistance, the translation of these advantages into measurable clinical benefits remains variable across studies. Differences in study design, treatment protocols, and patient characteristics may contribute to inconsistent findings [2,7]. Although SLBs alone have not consistently demonstrated superior clinical outcomes across all treatment parameters, recent clinical investigations suggest that their combination with adjunctive acceleration procedures may enhance treatment efficiency. A study conducted on the rate of orthodontic tooth movement demonstrated that the use of an SLB system combined with corticision significantly increased the rate of orthodontic tooth movement, highlighting the potential of integrating SLBs with minimally invasive acceleration techniques to optimize treatment outcomes [23].

The higher cost of SLBs and susceptibility to clip deformation or mechanical failure are additional practical limitations in routine clinical use. Digital technologies and AI are rapidly transforming orthodontic practice, creating new opportunities to amplify the advantages of SLBs. Digital workflows now encompass three-dimensional intraoral scanning, cone-beam computed tomography, computer-aided bracket positioning, treatment simulation, and patient-specific appliance fabrication [24]. AI-based algorithms are being developed to automate cephalometric analysis, predict tooth-movement trajectories, monitor treatment progression, and optimize biomechanical force systems [24,25]. The expanding role of AI across healthcare and medical education highlights its potential to enhance clinical decision-making, personalize treatment planning, improve efficiency, and support data-driven practice, including within orthodontics [26].

Limitations

This review has several limitations. First, as a narrative review, it does not employ the rigorous methodology of a systematic review or meta-analysis, making it susceptible to selection bias in the inclusion and interpretation of evidence. Second, many of the studies evaluating SLBs exhibit considerable heterogeneity in terms of study design, patient populations, bracket systems, archwire sequences, treatment protocols, and outcome measures, limiting direct comparisons and generalizability. Third, much of the evidence supporting friction reduction is derived from in vitro investigations that may not accurately replicate the complex biological and mechanical conditions of the oral environment. Additionally, several clinical studies included relatively small sample sizes and short follow-up periods, reducing the ability to assess long-term treatment stability, periodontal outcomes, and patient-reported benefits. Furthermore, many available studies have relatively short follow-up periods, limiting assessment of long-term treatment stability, periodontal outcomes, and patient-reported benefits.

Future directions

Future research should focus on conducting large-scale, multicenter randomized controlled trials with standardized treatment protocols and outcome measures to provide more definitive evidence regarding the clinical effectiveness of SLBs. Long-term studies evaluating treatment stability, root resorption, periodontal health, and patient-reported outcome measures are particularly needed. Further investigations should also explore the comparative performance of active, passive, and interactive self-ligating systems across different malocclusion types and treatment complexities. The integration of SLBs with digital orthodontic workflows, AI-based treatment planning, three-dimensional printing technologies, and remote monitoring systems represents a promising area for future development. Additionally, research into bioactive coatings, smart sensor-enabled brackets, and personalized orthodontic appliances may further enhance treatment efficiency, patient comfort, and precision-based orthodontic care. These advances have the potential to redefine the role of SLBs within contemporary evidence-based orthodontic practice.

## Conclusions

SLBs represent an important advancement in orthodontic appliance design, offering improved sliding mechanics, enhanced chairside efficiency, and favorable patient-centered outcomes. Current evidence most strongly supports benefits related to comfort, oral hygiene, and clinical handling, while further high-quality studies are needed to clarify their long-term clinical advantages. Future well-designed multicenter randomized trials with standardized protocols and long-term follow-up are needed to clarify the clinical significance of SLBs and their role in modern orthodontic practice. The integration of digital orthodontics and AI may further enhance their effectiveness and support more personalized treatment approaches.
